# Gut microbiome and aging-related disorders: mechanistic insights and emerging microbiome-targeted therapies for healthy aging

**DOI:** 10.3389/fnut.2026.1839461

**Published:** 2026-10-27

**Authors:** Md Minarul Islam, Gabsik Yang, Ki Sung Kang

**Affiliations:** 1 Department of Food Science and Biotechnology, Gachon University, Seongnam, Republic of Korea; 2 College of Korean Medicine, Gachon University, Seongnam, Republic of Korea

**Keywords:** aging, dysbiosis, epithelial barrier, gut microbiome, immunity

## Abstract

**Background:**

The human gut harbors a diverse and dense microbial community that maintains metabolic, immune, and intestinal homeostasis through a symbiotic relationship with the host. Aging is a complex biological process that involves alterations in the gut microbiome, including decreased microbial diversity and a decline in beneficial microbiota that produce useful metabolites, which impair intestinal barrier function and enhance chronic low-grade inflammation (inflammaging).

**Scope:**

This review explores current evidence on the interaction between aging and the gut microbiome, emphasizing its impact on age-associated diseases, such as frailty and sarcopenia, cardiovascular issues, type 2 diabetes, obesity, osteoporosis, and neurodegenerative conditions. It further delves into the distinct microbial profiles of centenarians and evaluates microbiome-targeted interventions, such as dietary approaches, probiotics, prebiotics, synbiotics, fecal microbiota transplants, caloric restriction, intermittent fasting, and pharmaceutical agents, by synthesizing evidence from both human and animal research to emphasize their potential in fostering healthy aging and increased longevity.

**Key findings:**

Evidence from human and preclinical studies suggests that alterations in the gut microbiome associated with aging facilitate immune dysregulation, metabolic dysfunction, and the progression of various chronic diseases. Whereas, centenarians possess distinctive microbial signatures rich in beneficial metabolites that could promote healthy aging and prolonged life. Additionally, microbiome-targeted interventions have demonstrated promise in enhancing gut microbial diversity and host wellbeing, though their clinical effectiveness needs additional validation.

**Conclusion:**

The gut microbiome is a potential focus for supporting healthy aging and warding off age-related illnesses. Nonetheless, its significant inter-individual variability emphasizes the necessity for personalized microbiome-based approaches, which require validation through extensive longitudinal studies and properly structured randomized controlled trials to demonstrate causality and verify long-term effectiveness.

## 1 Introduction

The aging global population is a major social and economic issue, prompting emphasis on research related to aging. Modern humans now live much longer than they did in the early 20th century due to advances in medicine and lifestyle improvements. Medical advancements have increased life expectancy, with projections indicating that over 1.6 billion people will be aged ≥65 years by 2050 (1). However, this demographic change has also led to an increase in age-related illnesses. If the factors that contribute to healthy aging are not addressed, healthcare costs are expected to increase. Despite longer lifespans, the period of disease-free life, known as the health span, lags by 9 years (2). Understanding the mechanisms of healthy aging and implementing interventions is essential to delay the onset of diseases and reduce their severity later in life.

Although aging is not considered a disease, it poses risk for several health disorders. Aging is a natural and inevitable process characterized by diminished regenerative capabilities of the body. As every living entity ages, the effects on physical and cognitive functions differ among individuals (3). This intricate biological process is fueled by cellular and molecular damage, including stem cell exhaustion, genetic instability, telomere attrition, cellular senescence, mitochondrial dysfunction, loss of protein homeostasis, epigenetic alterations, and disruption of nutrient sensing. Collectively, these factors lead to chronic inflammation and an increased susceptibility to a range of chronic diseases (4–6). Aging is a natural process that cannot be avoided; therefore, achieving healthy aging is a realistic and achievable goal. Healthy centenarians offer valuable insights for researchers studying aging. Genetic and non-genetic factors such as lifestyle choices and environmental conditions play a considerable role in aging. Therefore, adjusting for nongenetic factors can be an effective strategy for promoting healthy aging (7).

Mounting evidence suggests that the gut microbiome (GM) is a key etiological factor in the aging process (8). Human guts harbor a diverse community of microorganisms, including bacteria, fungi, archaea, and viruses, collectively known as GM. The GM of each person is distinctive, adaptive and comprises approximately 100 trillion microorganisms that are peculiar fingerprints (9). GM colonizes the body from birth, possibly even before, with the maternal vaginal, gut, and skin microbiota as important sources of these initial bacteria. After birth, the gut microbiota rapidly diversifies, leading to the development of a distinctive and functionally resilient microbiome for each individual that gradually changes (10). A well-balanced gut microbiota is essential for numerous aspects of human health, including nutrient absorption, immune regulation, epithelial barrier integrity, neurodevelopment, behavior, cognition, and stress response (11). Disruptions in the microbiome, termed dysbiosis, harm the host and are associated with age-related diseases and aging. Conversely, aging disrupts the delicate balance between the host and gut microbiota, leading to dysbiosis. However, the precise mechanisms underlying the pathophysiological alterations associated with age-related gut dysbiosis remain unclear. Clinical and preclinical studies have suggested strong associations between gut dysbiosis, persistent inflammation, and age-related diseases. Dysbiosis influences systemic inflammation, immune function, and metabolism, which are crucial aspects of aging (12). Hence, we speculate that targeting gut microbiota could be a novel approach to anti-aging.

Populations with long lifespans tend to have a diverse gut microbiota (13). Experiments conducted on progeroid mice, which mimic accelerated aging, have demonstrated that supplementation with specific strains of gut microbiota can extend their lifespan (14). Research suggests that interventions targeting the microbiome, such as probiotics, prebiotics, and synbiotic supplements, and dietary interventions, such as the Mediterranean diet, caloric restrictions, and regular exercise, can help maintain eubiotic gut health (15–20). These approaches support healthy aging by promoting a diverse and resilient GM. This growing body of evidence underscores the potential of microbiome-focused strategies in promoting longevity and addressing age-associated health challenges. Although evidence connects GM alterations to age-related changes, several key questions still need to be addressed. Limited longitudinal human studies, inconsistencies in methodologies, and the ambiguous causal link between gut dysbiosis and aging still hinder clinical application. This review explores the impact of GM on aging, age-associated conditions while highlighting significant research gaps and future directions for microbiome-targeted interventions to promote healthy aging.

## 2 Methodology

The PubMed, Scopus, Google Scholar, and Web of Science databases were searched for English-language articles published up until March 2026 in order to compile this narrative review. The search used keywords such as “gut microbiome,” “aging,” “healthy aging,” “age-related diseases,” “probiotics,” “prebiotics,” “synbiotics,” “microbial metabolites,” “sarcopenia,” “frailty,” “obesity,” “diabetes,” “cardiovascular disease,” “neurodegenerative disorders,” “osteoporosis,” and even “centenarians microbiome.” Articles that addressed age-related illnesses, healthy aging, the gut microbiome, microbiome-targeted therapies, or pertinent microbial metabolites and processes were included. Those were without pertinent mechanistic or clinical insight, or irrelevant the purview of this narrative review, were excluded. Since this was a narrative review, there was no rigorous systematic screening or PRISMA-guided approach; instead, the selection of studies was according to scientific merit and significance. By looking through the reference lists of chosen papers, more pertinent publications were found. The function of the gut microbiota in healthy aging and age-related illnesses was explored through preclinical and clinical research, with a focus on current high-quality findings and important mechanistic studies.

## 3 Association between GM and aging

### 3.1 Microbiome-mediated mechanisms influencing aging

The GM is dynamic and adaptable, forming a two-way relationship with the host to promote health. This intricate interaction between GM and aging has important implications for overall health and disease susceptibility (Figure 1).

**Figure 1:**
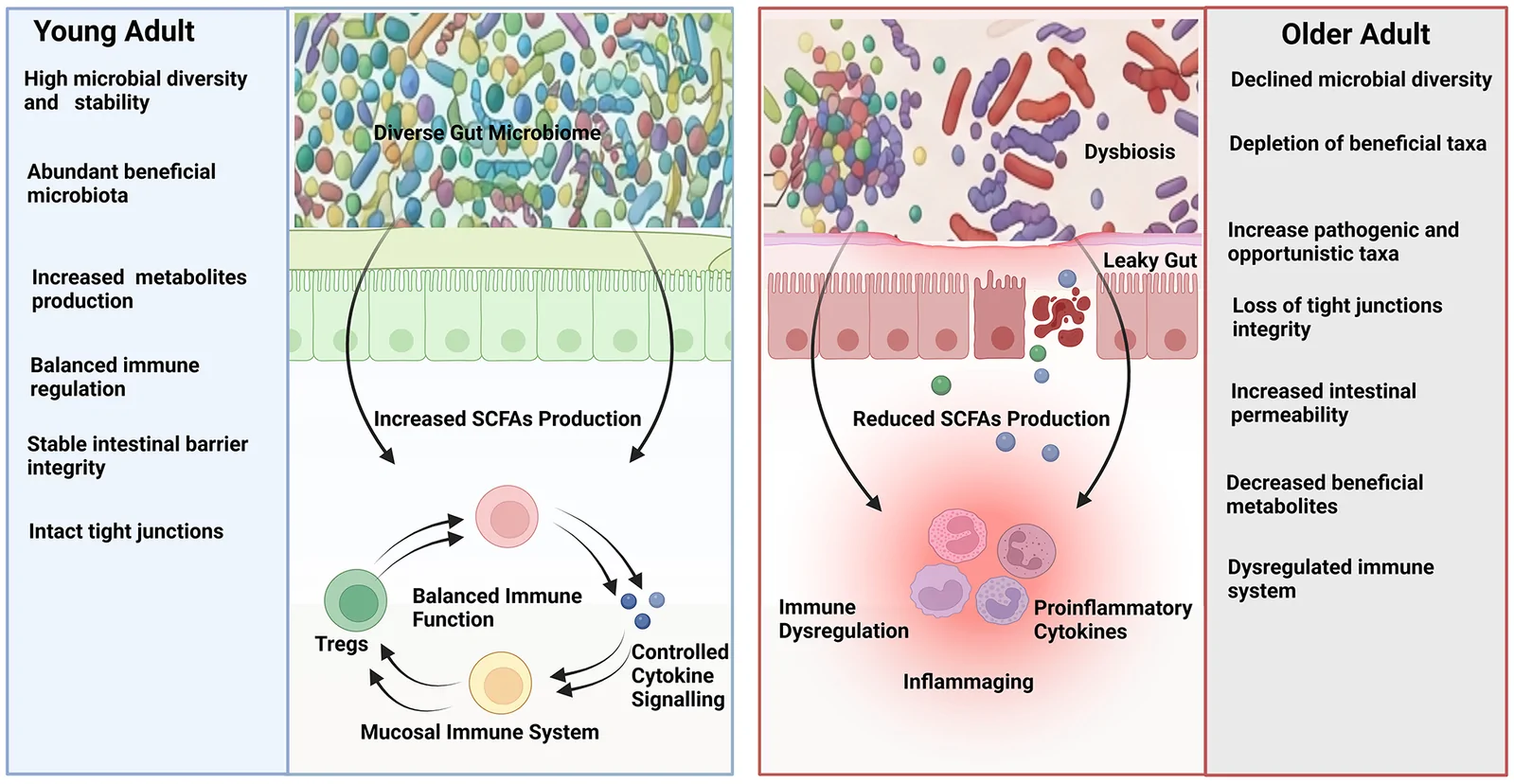
Distinctions between young and aged gut ecosystems. Young gut are rich with high microbial diversity, balanced microbial communities, and production of beneficial metabolites, including short-chain fatty acids. These microbial signature fosters a harmonious gut environment immune homeostasis, and overall health. In contrast, aging is associated with declined microbial diversity and gut dysbiosis, characterized by decreased SCFA production, elevated level of potentially harmful microbial products, increased intestinal permeability (“leaky gut”), and impaired immune regulation. These age-related microbiome alterations may contribute to systemic inflammation and the development of age-associated disorders (21). Created with biorender.com.

The immune system has evolved to work in harmony with various microorganisms to provide protection against pathogens and ensure tolerance to harmless antigens. Collaboration between the immune system and microbiota is essential for overall health. Aging affects the immune system differently in each individual (22), but generally results in a gradual decline in immune function, reduced adaptability, increased susceptibility to illness, and age-related dysbiosis (6, 23). Beneficial bacteria such as *Bifidobacteria, Akkermansia*, and *Lactobacilli* play a role in maintaining immune tolerance, whereas harmful bacteria such as *Enterobacteriaceae* and *Clostridium* contribute to infection and inflammation (24). This suggests that the host immune system plays a role in shaping the immune response and structure of the microbiome. A balanced GM is crucial for immune system development. The gut microbiota shapes the immune response of the body by producing bacterial components and metabolites. Germ-free mice exhibit impaired immune responses and are more susceptible to infections. The recolonization of germ-free mice with diverse microbiota restores immune function and pathogen resistance (25).

Gut bacteria are essential for food fermentation and producing vitamins (26). Interactions between the gut microbiota and dietary components can either harm or improve the gut barrier and lipid metabolism. As a result, the microbiota and its metabolites influence the intestinal barrier function, host metabolism, and pathogen colonization (27, 28). Short-chain fatty acids (SCFAs) are the primary products of the bacterial fermentation of dietary fibers and resistant starch in the colon. They play diverse roles in the gut and can affect distant organs, such as the brain and kidneys (29). Aging is associated with cognitive decline and reduced gut SCFAs levels. A study involving microbiome transfer from older to young germ-free mice indicated that the microbiome of elderly mice lowered SCFA levels, leading to behaviors resembling depression and memory deficits. This indicates that the aging microbiome can reduce SCFAs, leading to cognitive decline (30). SCFAs play vital roles in various physiological processes such as the proliferation of intestinal epithelial stem cells, hematopoiesis in the bone marrow, and intestinal epithelium integrity (31, 32). Additionally, SCFAs modulate immune responses by reducing proinflammatory cytokine production, improving microglial function, impeding and boosting B cell antibody production, and facilitating the differentiation and expansion of T regulatory cells (33–37). Alongside their anti-inflammatory effects, SCFAs, particularly butyrate, function as histone deacetylase (HDAC) inhibitors, influencing epigenetic regulation via enhanced histone acetylation. This encourages the expression of anti-inflammatory genes and inhibits pro-inflammatory signals, thus lowering inflammation associated with aging (38). Research has demonstrated that gut microbiota can generate or trigger neurotransmitter production, including serotonin, dopamine, γ-aminobutyric acid (GABA), norepinephrine, acetylcholine, and other neuroactive compounds. These substances have the potential to influence brain function and health (39). Additionally, the GM produces Trimethylamine N-oxide (TMAO) and phenylacetylglutamine (PAGln), which are associated with acute myocardial infarction and T2DM (40, 41). Beyond microbial metabolites, component including LPS, peptidoglycan, bacterial proteins like neurotoxins, and microbial small non-coding RNA can interact with host immune pathways and potentially promote inflammation, immunosenescence, and neuroinflammation, especially when their exposure is increased by age-related gut barrier dysfunction (42).

Age-related alterations in the gut bacteria can lead to intestinal issues, inflammation, and early death (43). Thevaranjan et al. demonstrated that age-related changes in the microbiota due to aging enhance intestinal permeability, elevate systemic inflammation, and hinder macrophage function. Germ-free mice were shielded from these effects, and low TNF-levels reversed the microbiota alterations. In aged mice, microbial elements also elevated interleukin-6 levels, which contribute to inflammation associated with aging (44). Age-related dysfunction of the epithelial tight junctions increases gut permeability (45). Loss of putative anion transporter-1 further disrupts the GM and its metabolites, resulting in compromised gut barrier integrity and increased susceptibility to intestinal inflammation in the host (46). However, further research is required to fully understand the mechanisms underlying the age-related increases in gut permeability.

### 3.2 GM dysbiosis affects the health of aging people

Over 2,000 years ago, Hippocrates stated that “all disease starts in the gut” (47). Over a century ago, Metchnikoff further developed this concept by proposing that health could be enhanced and aging could be delayed by altering the GM with the beneficial bacteria found in yogurt. Metchnikoff believed that ailments in older individuals were caused by harmful bacterial toxins leaking from the colon, causing phagocytes to attack healthy tissues (48). The importance of chronic inflammation in activating and impairing phagocytic cells has been confirmed over the centuries. As people age, there is a shift toward a less specific innate immune response, leading to a decline in adaptive immune function. This change presents challenges in meeting the healthcare requirements of older adults (49). GM constantly changes in response to factors such as diet, medication, physical activity, and social interactions. These factors affect the composition and function of the GM (50, 51). To adapt to these changes, the GM is adjusted by altering the bacterial species composition and metabolic activities. This adjustment is controlled by the host immune system, which promotes the growth of beneficial bacteria and eliminates harmful ones (52). Here, we present an overview of key alterations in microbiome composition and function associated with age-related illnesses (Figure 2).

**Figure 2:**
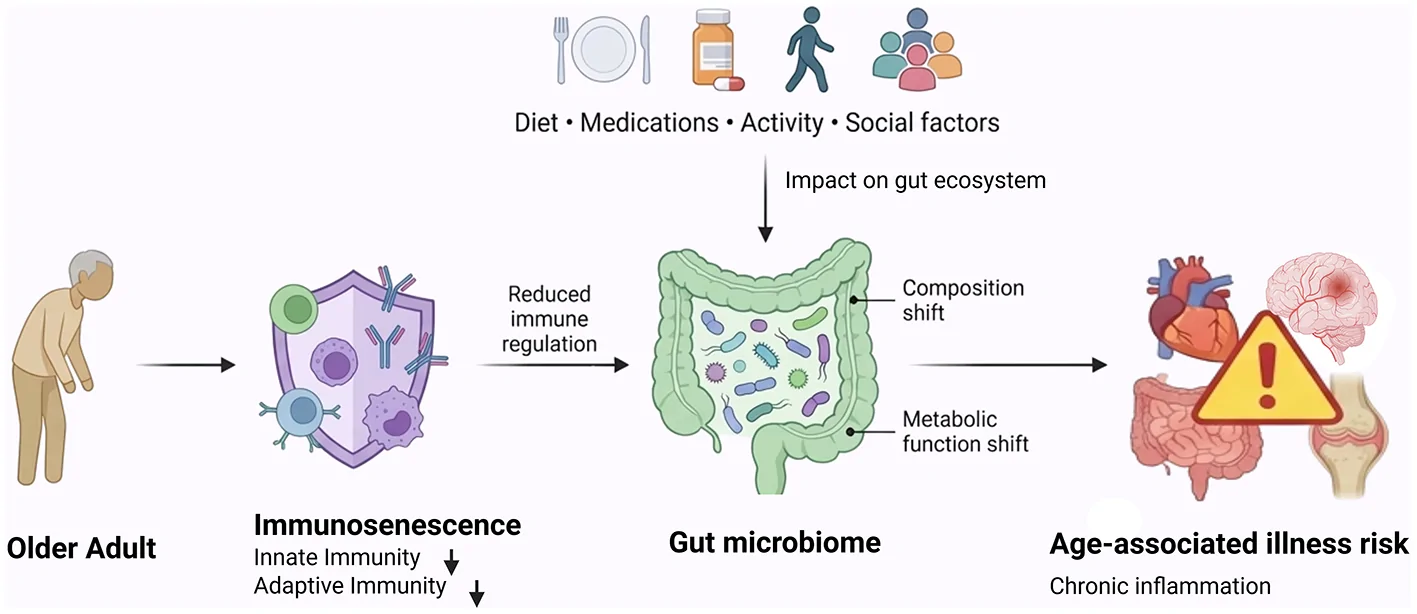
The figure illustrates the associated axes connecting environmental elements, gut microbial imbalance, and age-related immune decline, showcasing how the factors like nutrition, medication, exercise, and social aspects prompt compositional and metabolic changes in the intestinal environment that increase the risk of age-related diseases. Simultaneously, aging acts as a trigger for immunosenescence and decreased immune regulation, marked by specific functional declines in both innate and adaptive immunity (53). (↓Decrease) Created with biorender.com.

#### 3.2.1 Frailty syndrome and sarcopenia

Frailty is a prevalent clinical geriatric syndrome in older adults marked by heightened vulnerability and an increased risk of adverse health outcomes such as falls, disability, hospitalization, functional decline, dependence, and mortality (54). GM significantly affects patients with frailty syndrome. Aging alters the natural barrier of the gastrointestinal (GI) tract, allowing certain microorganisms and their by-products to enter the bloodstream and trigger inflammation. A study in older adults with physical frailty and sarcopenia, participants with physical frailty and sarcopenia exhibit higher levels of aspartic acid in their serum, lower levels of threonine and macrophage inflammatory protein 1α, and increased levels of *Oscillospira* and *Ruminococcus*, as well as decreased levels of *Barnesiellaceae* and *Christensenellaceae*, than those in the control group (55). This finding, confirmed by Cui et al., established a genetic correlation and causal association between a high abundance of *Christensenellaceae R-7* and reduced risk of frailty (56). In a study with 176 Korean older adults with frailty reported to have altered microbial diversity. Elevated level of harmful and reduced beneficial taxa are associated with frail individual. Notably, the presence of butyrate-producing *Coprococcus* is linked to lower rates of atopic disease, depression, and irritable bowel syndrome (57), whereas *B. fragilis* toxin triggers a pro-carcinogenic inflammatory response in colonic epithelial cells (58). A mendelian randomization (MR) study reveals genetic proof that certain gut microbial taxa could causally affect epigenetic age acceleration and frailty, suggesting a possible involvement of gut microbiota in aging-related diseases (59). Since factors like diet, medications, and environmental factors affect gut microbiota, further research is needed to clarify its causal role in frail older individual.

Sarcopenia also occurs as part of the normal aging process and is characterized by a gradual loss of skeletal muscle mass, quality, and strength. Sarcopenia can lead to physical frailty, increased risk of falls and fractures, reduced mobility, and low quality of life (60). The gut–muscle axis is essential for regulating age-related muscle health; however, the mechanisms underlying this axis remain poorly understood, and research findings are inconsistent. Observational studies indicate that gut microbiota can affect muscle mass and function by modulating inflammation, immunity, metabolism, endocrine function, and insulin sensitivity (61). One study found that certain components of the gut microbiota are linked to sarcopenia-related traits. *Alcaligenaceae, Family XIII*, and *Paraprevotella* were associated with an increased risk of low hand-grip strength, whereas *Streptococcaceae* showed a negative association. Actinomycetales, *Actinomycetaceae, Bacteroidaceae, Porphyromonadaceae, Prevotellaceae, Bacteroides, Marvinbryantia*, and *Phascolarctobacterium* are associated with a higher risk of appendicular lean mass (ALM) loss. In contrast, *Eubacterium fissicatena* is negatively associated with ALM (62). Altered gut microbiota can affect muscle mass and function through various mechanisms involving metabolites and the endocrine and immune systems. One study revealed disruptions in the GM and metabolome in individuals with sarcopenia, showing significant reductions in bacteria, including *Bifidobacterium longum, Bifidobacterium pseudocatenulatum*, and *Bifidobacterium adolescentis*, as well as in metabolites such as shikimic acid (63). The shikimic acid (SA) pathway is commonly used by bacteria to synthesize aromatic amino acids (L-tryptophan, L-phenylalanine, and L-tyrosine) and other secondary metabolites (64). For example, tryptophan stimulates insulin-like growth factor-1 production in muscle cells, leading to the upregulation of genes involved in myofibril formation (65). However, frailty and sarcopenia are complex issues influenced by various factors, making it challenging to establish causal relationships. Therefore, further randomized controlled trials (RCT) are needed to investigate the potential protective role of the GM against frailty and sarcopenia, to understand its underlying mechanisms, and to identify effective interventions to enhance the health and quality of life of older adults.

#### 3.2.2 Diabetes

Diabetes is a common metabolic disorder characterized by elevated blood sugar levels owing to problems with insulin secretion, insulin action, or both. This condition is prevalent in older adults (66). Diabetes mellitus accelerates aging through common pathophysiological mechanisms (67). Aging-related alterations in body composition, combined with overeating and poor diet, disrupt the gut microbiota. GM disruptions elevate intestinal permeability, enabling molecules such as lipopolysaccharides (LPS) from gram-negative bacteria to enter the bloodstream. Excess calories and LPS trigger chronic inflammation, activate the endocannabinoid system, and promote the accumulation of senescent cells and dysfunctional adipose tissues. This excessive build-up of adipose tissue can lead to insulin resistance and, ultimately, diabetes mellitus (68, 69).

Adults with type 2 diabetes (T2D) have a different gut microbiota composition than healthy individuals. Typically, *Bifidobacterium, Bacteroides, Faecalibacterium, Akkermansia*, and *Roseburia* are found in lower abundance in individuals with type 2 diabetes (T2D), while *Ruminococcus, Fusobacterium*, and *Blautia* are abundant. Whether these changes in the microbiota are the cause or result of T2D is unclear and further investigation is required (70). A 16S rDNA sequencing investigation of individuals with end-stage renal disease showed distinctive gut microbial profiles distinguishing diabetic nephropathy (DN) from non-diabetic nephropathy (NDN). Patients with DN showed higher levels of *Oscillibacter, Bilophila, UBA1819*, and various *Ruminococcaceae* taxa, indicating a possible association of gut dysbiosis in the progression of DN (71). Overall, T2D is linked to a decrease in beneficial SCFA-producing bacteria, especially those producing butyrate, which improves insulin sensitivity (72). SCFAs play a crucial role in promoting the secretion of glucagon-like peptide-1 by intestinal L-cells via specific G protein receptors (GPR41 and GPR43). This process significantly affects pancreatic function, insulin release, and central appetite regulation (73). Despite the notable correlation between gut dysbiosis and T2D, it is still unknown if this is a cause or an effect of the illness. Recent bidirectional MR studies indicate that gut microbial taxa may contribute to T2D susceptibility, while reverse MR revealed no causal relationship between T2D and gut microbiota (74). To fully understand the function of gut dysbiosis in aging and age-related illnesses, further research is required.

#### 3.2.3 Obesity

Obesity is associated with alterations in GM diversity. The ratio of Firmicutes to Bacteroidetes is increased in individuals with obesity. However, the consistency of this observation and its reliability as a biomarker remain unclear, which likely reflects methodological heterogeneity, as well as variations in lifestyle, dietary habits, and environmental factors (75, 76). In older adults, the gut microbiota tends to exhibit lower bacterial diversity, shifts in dominant species, decreased levels of beneficial microorganisms, increased levels of facultative anaerobic bacteria, and reduced levels of total short-chain fatty acids (57). This decline in gut microbiota diversity and function has been associated with an increased risk of immune metabolic conditions, such as obesity (77).

In older individuals (aged 55–85), obesity correlated with gut dysbiosis, marked by diminished microbial diversity, elevated *Bacteroides*, and lower levels of *Akkermansia* and *Christensenellaceae_R-7_group*, along with inferior cognitive performance, indicating a possible involvement of the gut–muscle–brain axis in cognitive aging (78). During aging, there was an increase in Firmicutes and Actinobacteria, and a decrease in Bacteroidetes and Tenericutes. This shift results in a higher Firmicutes-to-Bacteroidetes ratio and elevated levels of lipopolysaccharides (LPSs) (79), which are potent activators of Toll-like receptor 4 (TLR4). The increased levels of LPS, which contain lipid A, may translocate across the intestinal mucosa, possibly facilitated by the lipoproteins involved in triglyceride transport. This mechanism may contribute to persistent low-grade inflammation and insulin resistance commonly observed in obese individuals (80). Gut dysbiosis is consistently contribute to the pathophysiology of obesity; however, the ambiguity surrounding whether it is a cause or a result of obesity persists because of the intricate relationships between gut microbiota, diet, host genetics, metabolism, and environmental influences.

#### 3.2.4 Cardiovascular diseases (CVD) and hypertension

An imbalance in GM is associated with CVD and hypertension. However, the causal link between hypertension and gut microbiota dysbiosis is still unclear, and it is uncertain whether dysbiosis leads to or results from hypertension. Understanding its association with age-related CVD is crucial for effective prevention and treatment. The composition of the gut microbiota and metabolites, such as TMAO, bile acids (BAs), and SCFAs, plays a significant role in the development of these diseases through diverse molecular pathways, presenting novel therapeutic opportunities (81). Jie et al. compared the gut microbiota of 218 individuals with atherosclerotic cardiovascular disease (ACVD) to that of 187 healthy controls. The results showed that patients with ACVD had higher levels of harmful bacteria such as *Escherichia coli, Klebsiella spp., Streptococcus spp*., and *Eggerthella lenta*, while beneficial bacteria, such as *Roseburia intestinalis* and *Faecalibacterium prausnitzii*, were lower. Common gut microbiota such as *Bacteroides spp*. and *Prevotella copri* are also reduced in patients with ACVD, indicating significant microbial differences associated with the disease (82). SCFAs are crucial for regulating metabolic health and the immune system. They assist in the control of blood glucose and lipid metabolism, reduce oxidative stress, and exhibit anti-inflammatory and antitumor effects by modulating cytokine and chemokine secretion (83). SCFAs may protect against atherosclerosis and cardiovascular diseases. In atherosclerosis, a GM imbalance decreases butyrate-producing bacteria and increases intestinal permeability and lipopolysaccharide (LPS) levels (84, 85). TMAO is a product of dietary choline and l-carnitine metabolism by gut bacteria, has shown its role in inhibiting cholesterol metabolism and promoting platelet aggregation, thrombosis, and atherosclerosis. It plays a significant role in the pathogenesis of CVDs and is a crucial risk factor for the onset and prognosis of CVDs (86). Additionally, the gut–liver axis may play a role in age-related cardiometabolic conditions, as TMAO derived from gut microbiota has been linked to cardiovascular complications related to metabolic dysfunction-associated steatotic liver disease (MASLD) (87). Another gut microbial metabolite, phenylacetylglutamine, has been linked to an increased CVD risk. This metabolite is produced by gut bacteria from dietary proteins and can affect cardiovascular health by influencing various physiological pathways, underscoring the significant role of gut microbiota in CVD risk (88). Targeting the gut microbiota to reduce the production of metabolites such as TMAO and phenylacetylglutamine is a promising approach for treating CVD.

Both monocrotaline-induced pulmonary hypertension and patients with pulmonary hypertension exhibit disrupted gut microbiota-mediated metabolism of several amino acids, including arginine, proline, and ornithine (89). Dysregulation of arginine metabolism is a significant factor in the development of endothelial dysfunction and pulmonary hypertension in sickle cell disease and is closely linked to increased mortality in affected patients (90). This disruption is linked to an increased presence of bacteria, such as *Blautia, Bifidobacterium*, and *Collinsella*, which are known to promote the biosynthesis of these amino acids. In contrast, the reference cohort (healthy control) showed a higher abundance of butyrate- and propionate-producing bacteria, including *Coprococcus, Butyrivibrio, Lachnospiraceae, Eubacterium, Akkermansia*, and *Bacteroides* (89). Butyrate, a SCFA, lowers blood pressure (91). Nonetheless, in a study hypertensive individuals had lower gut microbiota diversity, higher levels of *Lactobacillus* and *Akkermansia*, and fewer butyrate-producing bacteria such as *Roseburia* and *Faecalibacterium* (92). *Lactobacillus* and *Akkermansia* are typically associated with protection against hypertension (93–95), this study found higher levels of these bacteria in individuals with high blood pressure. The discrepancies might indicate the constrained species and strain-level resolution of 16S rRNA sequencing, which frequently lacks taxonomic precision and accuracy to effectively assign species-level associative research for particular conditions. Moreover, differences in study cohorts, dietary patterns, drug usage, ethnic backgrounds, stages of disease, sample collection, and sequencing platform may be a key factor leading to inconsistent or non-reproducible results in microbiome studies. Therefore, shifts in the abundance of these genera must be interpreted with caution, as they could indicate ecological adaptations or compensatory reactions to gut dysbiosis instead of being direct factors contributing to diseases. Additional mechanistic and longitudinal research is required to elucidate their functions in hypertension.

#### 3.2.5 Osteoporosis

Osteoporosis is a progressive skeletal disorder that weakens the bones, reduces bone mass, and increases the risk of fractures. When the bone mineral density decreases or when the quality and structure of the bone changes osteoporosis occur. Older adults and postmenopausal women are at higher risk of this condition (96). The potential impact of GM on osteoporosis, particularly in older individuals, has shown that it could substantially affect bone metabolism and degenerative skeletal conditions.

A MR and single-cell analysis study found that Burkholderiales influence osteoporosis and osteoclast activity. Burkholderales increases the number of osteoclasts while potentially reducing the risk of osteoporosis, possibly through immune regulation and production of metabolites such as SCFAs. This suggests that balanced bone remodeling requires both osteoclasts and osteoblasts (97). A study of 60 patients with osteoporosis and 60 matched controls found higher levels of *Actinomyces, Eggerthella, Clostridium Cluster XIVa*, and *Lactobacillus* in the osteoporosis group, and a decrease in *Veillonella*. This reduction in *Veillonella* could affect equol production, and in turn, bone resorption (98). SCFAs play a role in bone metabolism by improving calcium absorption and reducing inflammation (99). SCFAs such as propionate and butyrate can increase bone density and prevent bone loss by altering osteoclast function and inhibiting bone breakdown without affecting bone formation. This is achieved by promoting glycolysis rather than oxidative phosphorylation in the osteoclasts, thereby suppressing the expression of key osteoclast genes (100). Immune dysregulation caused by gut dysbiosis may contribute to chronic inflammation, potentially leading to osteoporosis. Chen et al. proposed that targeting gut microbiota could be an effective treatment for osteoporosis. Microbiota affects bone metabolism by modulating immune reactions, particularly inflammation. SCFAs produced by microbiota, like butyrate and propionate, may potentially enhance the production of regulatory T cells (Tregs), resulting in anti-inflammatory responses (101). Sex-specific variations, especially the decline in estrogen following menopause, may modify gut microbiota composition and could play a role in osteoporosis and other age-related complications, highlighting the importance to account for sex-specific differences in microbiome-oriented therapies (102). The gut microbiota produces essential vitamins, including vitamins K and B, which are important for bone health and metabolism. Folate (vitamin B-9) and cobalamin (vitamin B-12) directly affect bone health. Low levels of these vitamins can increase homocysteine levels, impair collagen crosslinking, and affect osteoclast and osteoblast activities, leading to weakened bones and fractures (103). Vitamin K, synthesized by gut microbiota, plays a crucial role in maintaining bone matrix quality (104). Thus, for the crucial influence of gut microbiota on bone health, it has become a new therapeutic target for preventing bone loss and treating osteoporosis. However, there is limited knowledge regarding the causal link between GM dysbiosis and osteoporosis.

#### 3.2.6 Alzheimer's disease

Alzheimer's disease (AD), which is the most prevalent form of dementia, progressively affects memory, cognitive abilities, and daily activities. The gut microbiota in patients with AD differs from that in healthy individuals, potentially affecting disease development and progression. Aging, the main risk factor of AD, could alter the microbiota involved in cognitive decline (105). Changes in the gut microbiota have been associated with AD development, with potential underlying mechanisms. For instance, a study on elderly Korean patients with mild cognitive impairment (MCI) found a low relative abundance of *Prevotella, Coprococcus, Akkermansia, Lachnospiraceae UCG-010, Prevotellaceae UCG-001*, and *Clostridia UCG-014* in the MCI group. Conversely, the relative abundances of *Bacteroides, Rothia*, and *Rikenellaceae RC9* gut groups were significantly higher in the MCI group (106). Beneficial bacteria, such as *Prevotella* supports mucus production in the colonic mucosal layer. Decreased levels of *Prevotella* appear to affect mucus formation, potentially leading to cognitive impairment. Lower levels of *Akkermansia* are associated with a compromised intestinal barrier, increasing the risk of AD, whereas elevated levels of *Bacteroides* may contribute to the translocation of lipopolysaccharides to the brain, which could worsen AD pathology (106). Grabrucker et al. demonstrated a gut–brain link in AD by transferring fecal microbiota from patients to rats. Recipient rats showed memory and mood impairments, as well as metabolic changes in the gut and brain. Additionally, serum from patients suppresses neurogenesis in human cell cultures, correlating with cognitive function and specific gut microorganisms (107). Recent studies have indicated that gut dysbiosis may contribute to AD development by promoting amyloid-beta aggregation, neuroinflammation, oxidative stress, and insulin resistance (108). Microbiota-related products and systemic inflammation have been linked, which leads to brain amyloidosis through endothelial dysfunction. SCFAs and LPS have been identified as potential links between gut microbiota and AD pathology (109). Recent MR research showed that particular gut microbial taxa may have a causal impact on the likelihood of developing AD, reinforcing the gut microbiota's involvement in AD pathogenesis. Nevertheless, current evidences do not indicate a noteworthy reverse causal relationship of AD on the makeup of these particular gut microbial groups (110, 111). Regardless of these results, the causal relationship between general gut microbiota dysbiosis in AD and the mechanisms involved has yet to be completely clarified.

#### 3.2.7 Parkinson's disease

Parkinson's disease (PD) is characterized by the abnormal accumulation of a protein called α-synuclein, which typically occurs in a folded state within neurons. The relationship between the gut and brain has led to speculation regarding the potential role of the GM in the development of PD, a complex condition that typically emerges later in life (112).

Gut dysbiosis exacerbates PD progression by promoting α-synuclein-mediated motor dysfunction. Gut bacterium *E. coli* produces an amyloid protein called curli, which accelerates α-synuclein aggregation and exacerbates PD pathology and behavioral deficits in mice (113). Several studies have investigated GM in PD. However, a definitive understanding of the specific microbiota associated with PD remains lacking. In particular, the microbiome of patients with PD showed elevated levels of *Clostridium cluster IV, Akkermansia, Bifidobacterium*, and lactic acid bacteria, while the levels of *Lachnospiraceae* and *Faecalibacterium* decreased compared to those in the control group (114, 115). Similarly, *Akkermansia, Oscillospira*, another SCFA-producing microorganism that degrades mucin glycans, are consistently enriched in patients with PD (116). PD is frequently linked to constipation and low BMI, and the abundance of *Oscillospira* is exacerbated in patients with constipation and low BMI (117). Stool samples from patients with PD showed elevated levels of mucin-degrading Verrucomicrobiae and LPS-producing Gammaproteobacteria compared with healthy controls. LPS is associated with α-synuclein aggregation and dopaminergic neuron loss, while α-Syn aggregation plays a central role in the initiation and progression of PD pathology (118). Studies indicate that α-synuclein aggregates in the enteric nervous system and can spread to the brain through the vagal nerve (119). A recent study indicated that *Enterobacteriaceae* may facilitate α-synuclein aggregation. In *Escherichia coli K-12*, nitrate reduction leads to nitrite formation, resulting in an oxidizing atmosphere that promotes dopamine-derived quinones and α-synuclein aggregation. Experiments in enteroendocrine STC-1 cells have demonstrated that nitrite, unlike nitrate, triggers α-synuclein aggregation (120). In addition, MR studies reveal a bidirectional link between gut microbiota and PD. Certain gut microbial taxa and metabolites from the microbiota might causally impact the risk of PD and its age of onset, while reverse MR analyses indicate that a genetic tendency toward PD may change the composition of gut microbiota (121–123). Nonetheless, the current evidence remains inconclusive and requires additional validation.

## 4 Microbiome-focused strategies for improving elderly health

Microbiome-targeted interventions can help regulate aging. Factors such as diet, exercise, and medication can influence intestinal microbiota (Figure 3). GM is closely associated with the health of older individuals, making it a promising avenue for promoting healthy aging. Human GM is known for its functional versatility; however, how effective therapeutic interventions can modulate this ecosystem, particularly in older individuals, is unclear. This uncertainty stems from the lack of understanding of how interventions affect core microbial taxa at the ecological level. Recent studies have explored methods to restore healthy microbiomes in aging populations to improve health and longevity.

**Figure 3:**
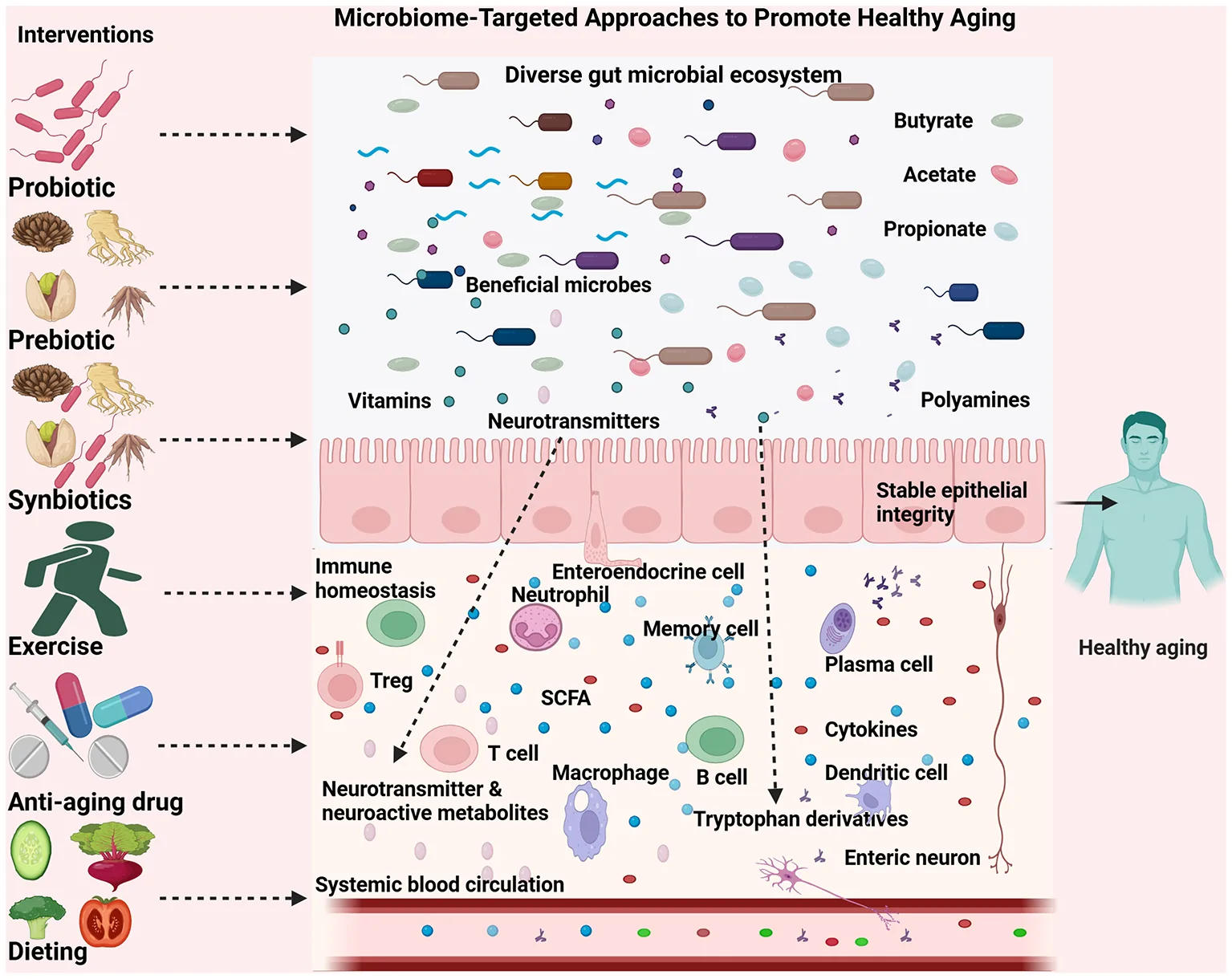
Impact of microbiome-focused intervention on the aging gut. Interventions targeting the microbiome could improve gut health in aging individuals by increasing microbial diversity and enhancing beneficial compound production, such as short-chain fatty acids (SCFAs), vitamins, neurotransmitters, and indole derivatives. Strategies such as probiotics, prebiotics, synbiotics, exercise, anti-aging medications, and dietary supplement help maintain gut integrity, promote anti-inflammatory cytokines, and reduce pro-inflammatory cytokines. Pro-gut strategies also modulate the gut–brain axis through neurotransmitter synthesis and vagus nerve function, thereby promoting healthy aging (124). Created with biorender.com.

### 4.1 Probiotics, prebiotics, and synbiotics

Bacteriotherapy using probiotics, prebiotics, and synbiotics help prevent and manage diseases. These interventions are cost-effective and easily accessible, promote balanced gut flora, and support healthy aging.

Probiotics represent an important intervention in aging research. Table 1 summarizes the functions of probiotic and non-probiotic bacteria and their metabolites in aging. Bowel movements are essential for life. Atypical bowel movements result in constipation. Elderly individuals are more prone to encounter this condition. Elderly people with functional constipation undergoing treatment with probiotic combinations experience an increase in stool frequency without experiencing any side effects (144). However, it was a single-center study with a limited number of participants; to validate the probiotic mixture's effect on stool frequency and transit time, additional long-term, multicenter, randomized, controlled trials are required. During aging, skin experiences transformations that lead to diminished elasticity, increased fragility, and altered immune response, ultimately resulting in chronic dermatological conditions. *Streptococcus thermophilus* delay skin aging and improves skin health by promoting skin cell proliferation, increasing collagen production, protecting against DNA damage, and inhibiting hyaluronidase (145). Obesity and aging are interrelated public health issues, with prevalence increasing with age. Treatment with *Bifidobacterium breve B-3* in pre-obese adults appear to reduce body fat without increasing visceral fat and improve triglyceride and HDL levels, indicating its potential as a safe strategy for body fat reduction (146). A study reported that more than 6 weeks continuous supplementation with *Lactobacillus plantarum TWK10* led to significant enhancements in muscle mass, strength, and endurance in elderly individuals. These improvements were pronounced at higher doses, which effectively reduced sarcopenia and physical frailty (147). Older individuals with cognitive disorders showed improved cognitive performance and elevated serum levels of brain-derived neurotrophic factor when treated separately with *Lactobacillus rhamnosus, Lactobacillus plantarum*, and *Bifidobacterium longum*, compared to a placebo (148, 149). Probiotic *A. muciniphila* reduces inflammation in the body and atherosclerotic lesions, leading to decreased macrophage infiltration and proinflammatory cytokines and chemokine expression, which is linked to lower levels of metabolic endotoxemia driven by improved intestinal barrier function and reduced gut permeability. However, the infusion of endotoxins could reverse the long-term protective effects of *A. muciniphila* against atherosclerosis (150). In patients with colon cancer, the probiotics *Bifidobacterium lactis Bl-04* and *Lactobacillus acidophilus* NCFM alter microbiota composition. The probiotic increases the abundance of butyrate-producing bacteria while potentially decreasing the levels of CRC-associated genera such as *Fusobacterium* and *Peptostreptococcus* (151). A blend of *Lactobacillus paracasei DSM 13434, Lactobacillus plantarum* DSM 15312, and *Lactobacillus plantarum DSM 15313*, along with individual supplementation of *Lactobacillus reuteri* ATCC PTA 6475 and *Bacillus subtilis* C-3102, showed positive outcomes in postmenopausal women with osteoporosis. These probiotics improve bone mineral density by modulating bone metabolism and altering the composition of the GM composition (152–154). Probiotics assist obesity and T2D management. A 6-month research project in Greece using the multi-strain probiotic LactoLevureR demonstrated notable decreases in HbA1c, fasting blood sugar, and overall cholesterol levels. Probiotic-modified gut microbial genera and metabolites are associated with enhanced glycemic control and metabolic wellbeing (155). *Akkermansia muciniphila* has surfaced as a possible marker of healthy aging because of its link to intestinal barrier strength, metabolic control, and decreased inflammation. Supplementation with pasteurized *A. muciniphila* in obese, insulin-resistant individuals enhanced insulin sensitivity, reduced insulin and total cholesterol levels, slightly decreased body weight and fat mass, and lowered liver dysfunction and inflammation markers, suggesting potential metabolic health advantages (156). Despite these encouraging results, varying diagnostic criteria across studies limit the draw to make conclusive judgments about the effectiveness of these strategies. Future research needs to address these methodological shortcomings to produce more trustworthy evidence and create practical, feasible, and clinically relevant strategies.

**Table 1:** Role of probiotic and non-probiotic bacteria and their metabolites in aging.

Microbial taxa/metabolites	Major functions	Potential aging-related benefits	Evidence level
*Lactiplantibacillus plantarum OL3246*	Anti-inflammatory, microbiota modulation	Reduced intestinal inflammation, improved gut health	Pilot clinical trial (125)
*Lactobacillus* capsule *(Lactobacillus rhamnosus Lactobacillus plantarum Lactobacillus paracasei Lactobacillus bulgaricus Lactococcus lactis)*	Anti-inflammatory, microbiota modulation	Enhanced grip strength, diminished inflammation, elevated testosterone	RCT (126)
*Lactobacillus rhamnosus GG*	Gut-brain axis regulation	Improved cognitive function	RCT (127)
*Bifidobacterium bifidum BGN4* and *Bifidobacterium longum BORI*	Microbiota and immune regulation	Reduced pro-inflammatory bacteria and immune activation	RCT (128)
*Akkermansia muciniphila*	Myostatin inhibition	Developed muscle strength	RCT (129)
*Lactobacillus rhamnosus + Bifidobacterium lactis*	Gut-brain axis regulation	Improved cognition, memory, and mood	RCT (130)
*Faecalibacterium prausnitzii*	Butyrate production, ferroptosis regulation	Attenuated age-related heart failure	Preclinical (131)
*Anaerobutyricum soehngenii*	Butyrate production, metabolic regulation	Improved glycemic control and cardiometabolic health	RCT (132)
*Roseburia intestinalis*	Gut barrier protection, epigenetic regulation	Improved learning and memory	Preclinical (133)
*Christensenella minuta DSM 22607*	Anti-inflammatory, gut barrier modulation	Reduced inflammaging and improved metabolic health	Preclinical (134)
*Parabacteroides distasonis*	Anti-inflammatory, mitochondrial modulation	Improved metabolic health and muscle function	Preclinical (135)
Butyrate, acetate, propionate	Immune regulation, anti-inflammatory	Reduced inflammaging and lung injury	Preclinical (136)
Urolithin A	Mitophagy activation, mitochondrial function	Improved muscle endurance	RCT (137)
Urolithin A	Mitophagy activation, immune modulation	Improved immune function and reduced inflammaging	RCT (138)
Butyrate	Mitochondrial support, gut barrier protection	Delayed multimorbidity and extended lifespan	Preclinical (139)
Indole-3-propionic acid	Antioxidant, anti-senescence	Improved musculoskeletal health and longevity	Preclinical (140)
Acetate	Anti-inflammatory, vascular protection	Improved arterial function	Preclinical (141)
Equol	Estrogen-like activity, metabolic modulation	Improved climacteric symptoms and potential skin aging benefits	RCT (142)
Bile acids	Neuroimmune regulation, microglial modulation	Improved neuroinflammation and cognitive function	Preclinical (143)

Probiotics are usually deemed safe for healthy adults, with a long history of use and minimal serious adverse events reported in clinical research. Nevertheless, safety data continue to be reported inconsistently, and potential hazards include gastrointestinal issues, elevated immune stimulation, and, on rare occasions, systemic infections, especially in at-risk populations (157, 158). Additionally, Individual responses to the same probiotic strain can differ substantially. Therefore, careful observation and additional well-designed research and personalized probiotic formulations are required to more accurately determine the long-term safety of probiotics in elderly individuals.

Prebiotics are defined as “a substrate that is selectively utilized by host microorganisms, conferring a health benefit (159).” These components stimulate the growth and metabolism of beneficial gut microorganisms, thereby improving health. Prebiotic supplementation, branched-chain amino acids and resistance exercise promote the abundance of beneficial *Bifidobacterium* and significantly improve the cognitive functions (160). Fructooligosaccharides have been shown to improve gut health by increasing *Bifidobacterium* and *Lactobacillus* levels, increasing overall SCFA levels, reducing alpha diversity, and influencing microbial communities, with different effects on specific taxa across age groups ranging from 25 to 70 years (161). In a RCT involving 74 adults aged 40–85 years with mild-to-moderate PD, supplementation with resistant starch enhanced *Faecalibacterium* levels and short-chain fatty acid production, lowered inflammation-related taxa, and benefited motor symptoms, non-motor symptoms, and overall quality of life (162). Eight weeks of yeast β-1,3/1,6-D-glucan (Wellmune) supplementation significantly improved insulin resistance, reduced TNF-α, and increased beneficial bile acids in adults aged 18–85 years with T2DM although these effects occurred without significant alterations in gut microbiota composition or short-chain fatty acid levels (163). Theou et al. discovered that taking Darmocare Pre^®^ (containing inulin and fructooligosaccharides) daily for 13 weeks led to a significant decrease in frailty levels compared to a placebo (maltodextrin) (164). Aging is associated with anabolic resistance and decreased microbiota diversity, which affect skeletal muscle mass and function. Older adults who combine prebiotics and protein supplements achieve greater gains in muscle strength than those who use proteins alone. This indicates that altering the GM could help combat anabolic resistance and potentially reduce sarcopenia and frailty in elderly (165).

The combination of probiotics and prebiotics, known as synbiotics, has a synergistic effect on gut health and benefits the host. Recent studies have explored the effects of synbiotic combinations on aging. In a 4-week randomized, double-blind, placebo-controlled crossover study involving 43 older volunteers, a synbiotic (*Bifidobacterium longum* and inulin-based Synergy 1) was found to enhance gut bacteria composition, boost butyrate production, and decrease the pro-inflammatory cytokine TNF-α (166). Synbiotic supplementation (*Lactobacillus sporogenes GBI-30*, maltodextrin, and fructooligosaccharide) administered to T1DM patients showed significant improvements in HbA1c, hs-CRP, insulin levels, and total antioxidant capacity compared to the placebo group. Fasting blood glucose levels also significantly decreased in the intervention group (167). Another synbiotic formula containing *Lactobacillus plantarum PBS067, Lactobacillus acidophilus PBS066*, and *Lactobacillus reuteri PBS072*, along with active prebiotics resulted in notable enhancements in waist circumference, fasting plasma insulin, cholesterol levels, inflammatory markers, and blood pressure in elderly patients (168). In addition, age-related increases in inflammation and oxidative stress may be reduced by the synbiotic formulations. In a 12-week RCT of patients with PD aged 50–80 years, synbiotic supplementation increased antioxidant capacity, decreased oxidative stress (lower Malondialdehyde and oxidative stress index), relieved depression, and improved quality of life and cognitive function, indicating its potential as an adjunct therapy for PD (169). However, the development of reliable therapies like probiotics, prebiotics, and synbiotics is complicated by the multifactorial pathophysiology of neurodegenerative illnesses, which involves genetic, mitochondrial, and gut–brain axis changes. In another study, synbiotic containing *Lactobacillus* and *Bifidobacterium* along with galactooligosaccharides, enhanced the gut microbiota diversity during weight loss. Increased *Lactobacillus* levels are linked to lower blood sugar levels, whereas diminished *Bifidobacterium* levels are correlated with reduced body weight and BMI (170). However, clinical studies on synbiotics and other biotic therapies are limited. Their potential in aging management is promising and warrants further investigation.

### 4.2 Fecal microbiota transplantation (FMT)

FMT is an emerging experimental strategy for rejuvenating gut microbial homeostasis through the transfer of healthy donor microbiota to a recipient. FMT holds potential to improve natural aging-related disorders through gut microbiota remodeling. Clinical evidences have demonstrated that FMT in older recipients improves gut microbial diversity, and ameliorates age-related impairments in cognition, immune function, and metabolism. A pilot study showed that older adults (age range, 63–90 years) suffering from dementia and severe *Clostridioides difficile* infection (CDI) who underwent FMT treatment showed substantial improvements in cognitive function compared to those treated with antibiotics, accompanied by alterations in gut microbial diversity and metabolic pathways, implying a potential involvement of the gut–brain axis (171). In another study, FMT showed moderate effectiveness in managing refractory CDI among hospitalized older individuals (ages 60–96 years), re-establishing gut microbial balance and lowering inflammatory markers (172). In addition, a case review of 31 adults aged 65 and older with *Clostridioides difficile* infection (CDI) revealed that FMT was both safe and effective, resulting in an 87% cure rate without any serious adverse events linked to FMT. These results potentially endorse the practicality of FMT in elderly individuals and its promise for microbiome-focused treatments in aging (173). In a retrospective analysis involving 172 adults over 60 years old with sarcopenia, FMT combined with resistance training enhanced muscle mass, strength, physical performance, and inflammation markers more effectively than resistance training alone. Mechanistically, FMT could improve muscle function by reestablishing gut microbial balance, lowering chronic inflammation, and influencing the gut–muscle connection (174). Additionally, a current double-blind, randomized, placebo-controlled clinical trial (ClinicalTrials.gov NCT06649981) is assessing the safety and effectiveness of oral FMT capsules from young, active donors in individuals aged 65–84 years. The research seeks to establish if enhancing gut microbial diversity can boost muscle strength, functional independence, cognitive abilities, and metabolic health by influencing the gut microbiota. The results are anticipated to offer significant clinical proof concerning the capability of FMT as a microbiome-centered approach for promoting healthy aging (175). Although FMT is approved by the U.S. Food and Drug Administration (FDA) for the treatment of recurrent CDI (176), currently its application in aging population is rare. Most clinical evidence is constrained by small sample sizes, and therefore, more extensive RCTs are necessary to validate its long-term effectiveness and safety in aging populations. In addition, FMT is generally regarded as safe; the reported adverse effects, such as diarrhea, bloating, flatulence, and abdominal discomfort, are moderate and temporary (177). However, particularly in immunocompromised and other high-risk persons, major problems and the spread of infectious organisms are still possible (178). To ensure the safety of FMT in elderly and vulnerable populations, careful donor screening, recipient selection, personalized FMT strategies and monitoring are imperative.

### 4.3 Diet, medication, and exercise promote healthy aging via GM modulation

Diet is the key to shaping the gut microbiota and supporting healthy aging. High-fiber intake through targeted dietary changes can quickly adjust nutrient levels, thereby altering microbiota composition to improve richness and diversity. The NU-AGE project implemented a 1-year Mediterranean diet intervention with elderly participants from five countries. Diet results in favorable alterations in the gut microbiota, including increases in beneficial species and decreases in harmful species associated with T2DM and colon cancer. These changes in microbiota are correlated with decreased frailty, enhanced cognitive function, and reduced inflammation, promoting healthy aging (179). A study of postmenopausal Mexican women found that a healthy diet was associated with reduced risk of metabolic syndrome (180). High dietary fiber intake is associated with changes in the composition of the GM, leading to an increase in beneficial bacteria, such as *Faecalibacterium, Lachnospira, Lactobacillus spp*., and *Bifidobacterium spp*., while reducing the levels of *Actinomyces, Odoribacter, and Oscillospira*, suggesting a potential role of dietary fiber in colorectal cancer through gut microbiota modulation (181). Consumption of soluble fiber is linked to higher levels of butyrate-producing bacteria, resulting in a reduction in proinflammatory bacteria (182).

Calorie restriction (CR) and dietary restriction (DR) involve reducing calorie intake without causing malnutrition to slow aging and improve health. CR affects the GM, immune system, and metabolism; promotes microbial diversity; reduces body fat; and enhances glucose tolerance. The microbiota from individuals practicing CR, when transplanted into germ-free mice, showed improved microbial diversity and immune cell profiles and reduced immune senescence. In mice, long-term CR preserves the young colon transcriptome, modifies the fecal metabolome, and prevents age-related changes in the microbiota (19, 183). In AD models, CR normalized age-related microbiome changes and reduced amyloid-beta deposition, especially in females, by reversing inflammatory gut profiles (184). A study involving 41 non-obese individuals who underwent a 3-week 40% CR program showed reductions in BMI, with a greater loss in the *Prevotella*-dominated (ETP) group than in the *Bacteroide*-dominated (ETB) group. Although CR had minimal effects on the gut microbial composition, it significantly increased blood 3-methylhistidine levels, indicating muscle protein turnover. The study found that baseline levels of seven microbial species were better predictors of BMI loss than amino acid levels, suggesting their potential for personalized nutrition (185).

In addition, intermittent fasting (IF) is an effective strategy for weight control and reducing the risk of obesity and diabetes. Li et al. (186) found that every-other-day fasting (EODF) can promote beige fat development, which helps in obesity, insulin resistance, and hepatic steatosis by altering the gut microbiota to increase acetate and lactate production and boost monocarboxylate transporter 1 in beige cells. Transplantation of the microbiota from EODF-treated mice into microbiota-depleted mice also led to white adipose tissue browning and improved metabolic health. In a study involving 45 individuals, IF reduced body weight and improved lipid profiles by increasing the levels of high-density lipoproteins and decreasing those of total cholesterol, triglycerides, and LDL/VLDL. IF also increases gut microbiota diversity and promotes beneficial bacteria, such as *Lactobacillus* and *Bifidobacterium*, which enhance metabolic health (187).

Certain drugs commonly prescribed to the elderly, such as proton pump inhibitors, metformin, antidepressants, laxatives, and statins, which modulate the GM in animal models, affect overall health (Table 2). Understanding the role of the microbiome in drug efficacy is essential for advancing precision medicine. The interaction between gut flora and non-antibiotic drugs is two-way. Drugs can alter the GM, which can influence drug responses by altering the enzyme structure. This can influence the effectiveness, activity, and potential adverse effects of a drug. Oral drugs undergo metabolism in the intestine before entering the bloodstream, where they interact with the gut microorganisms residing in the intestinal epithelium. Drug use can affect the composition of gut flora in various ways, and two main mechanisms have been proposed. The first mechanism suggests that drugs can lead to the migration of microbiota from other parts of the body to the gut (201). For example, proton pump inhibitors (PPIs) disrupt the gastric acid barrier, allowing external bacteria, particularly acid-resistant strains, to easily enter the gastrointestinal tract (202). The second mechanism, which may be more significant, involves drugs that alter the intestinal microenvironment and directly influence bacterial growth (201). Studies have shown that metformin promotes the growth of bacteria that produce short-chain fatty acids in the gut, ultimately contributing to its therapeutic effects in improving insulin resistance and glucose regulation (203).

**Table 2:** Antiaging drug-based gut microbiome alterations and effects on overall health.

Drug	Drug class	Study subject/evidence level	Mechanism of GM modulation	Direction of effect	Health outcome
Metformin	Biguanides (antidiabetic agent)	Human (RCT) (188)	Increases *A. muciniphila*, decreases *Romboutsia spp.*, and alters SCFA and amino acid (arginine/proline/glutamate) metabolic pathways	*↑ A. muciniphila and microbial metabolic pathways; ↓ Romboutsia spp*	Healthy aging
		Human (RCT) (189)	Modulates gut microbiota by increasing *Escherichia* and *Ruminococcus*, reducing *Intestinibacter* and *Roseburia*, and promoting SCFA production and glucose metabolism pathways	*↑ Escherichia coli, ↑ Ruminococcus torques, ↓ Intestinibacter bartlettii, ↓ Roseburia spp.;* ↑ acetate, butyrate, and valerate production	Healthy aging
Rapamycin	mTOR inhibitor (macrolide immunosuppressant)	Mice (preclinical) (190)	Triggers autophagy through mTOR suppression and alters gut microbiota by boosting beneficial species, especially *Lactobacillus reuteri*, while diminishing bacteria linked to inflammation	*↑ Lactobacillus spp., Lactobacillus reuteri, Prevotellaceae, Paraprevotella, Christensenella; ↓ Bacteroides sartorii, Peptostreptococcaceae, and Romboutsia*	Extenuate colitis
		Mice (preclinical) (191)	Alters gut microbiome composition by changing the Bacteroidetes/Firmicutes ratio and improving amino acid availability in the gut, aiding in immune regulation	↑ Firmicutes/Bacteroides ratio modulation and ↑ microbial amino acid availability (gut metabolites)	Anti-aging effects
Resveratrol	Polyphenolic compound	Mice (preclinical) (192)	Modulates gut–lung microbiota by enriching beneficial bacteria (*Akkermansia muciniphila* and *Bacteroides acidifaciens*), elevating butyrate production, and reducing LPS biosynthesis	*↑ Akkermansia muciniphila, ↑ Bacteroides acidifaciens, ↑ butyrate; ↓ LPS biosynthesis*	Alleviated allergic asthma and reduced inflammation
		Piglet (preclinical) (193)	Stimulate beneficial gut microbiota by boosting butyrate-producing *Roseburia* and elevating butyrate levels while diminishing potentially harmful bacteria	*↑ Roseburia, ↑ butyrate; ↓ Bacteroides and unidentified Enterobacteriaceae*	Gut homeostasis
Aspirin	Nonsteroidal anti-inflammatory drug (NSAID)	Human (RCT) (194)	Increases beneficial taxa (such as *Akkermansia* and *Ruminococcaceae)* and modifies bacteria linked to colorectal cancer risk to modify the composition of the gut microbiota	*↑ Akkermansia, Prevotella, Ruminococcaceae, Clostridium XIVa; ↓ Parabacteroides, Bacteroides, Dorea*	Prevent colorectal cancer in aging individuals (50–75 years)
		Mice (preclinical) (195)	Aspirin alters microbiota by elevating beneficial bacteria and decreasing potentially harmful taxa, while gut flora affect aspirin bioavailability through microbial breakdown	*↑ Bifidobacterium and Lactobacillus; ↓ Alistipes finegoldii and Bacteroides fragilis; ↑ aspirin degradation by Lysinibacillus sphaericus*	Prevent colorectal cancer
Spermidine	Polyamine compound	Mice (preclinical) (196)	Improves intestinal barrier integrity, lowers metabolic endotoxemia, and enriches the SCFA-producing *Lachnospiraceae NK4A136* group to reshape the gut microbiota	*↑ Lachnospiraceae NK4A136 group* and SCFA production*; ↓* metabolic endotoxemia and obesity-associated microbial alterations	Improve metabolic function
Senolytics	Senolytic agents	Mice (preclinical) (197)	Modifies age-related gut microbiota profiles while reducing intestinal senescence and inflammation	↓ senescence markers (p16Ink4a, p21Cip1) and inflammatory markers; alters age-related microbial composition	Anti-aging effects
Acarbose	α-Glucosidase inhibitor (Anti-diabetic agent)	Mice (preclinical) (198)	Increases beneficial microbial diversity and controls the Th17/Treg balance to alter the makeup of the gut microbiota and intestinal immune responses	↑ microbial diversity and richness; *↑ Oscillospira, Desulfovibrio*, and *Ruminococcus; ↓* arthritis-associated microbial alterations	Anti-inflammatory effects
		Human (RCT) (199)	Modulates SCFA-associated bacterial populations and enriches beneficial *Lactobacillus, Faecalibacterium*, and *Dialister* species to reshape gut microbiota	*↑ Lactobacillus, Faecalibacterium*, and *Dialister; ↓ Butyricicoccus, Phascolarctobacterium*, and *Ruminococcus*	Improve metabolic function
		Human (Multicentre clinical trial) (200)	Increases beneficial bacteria (*Lactobacillus* and *Bifidobacterium*) and modifies microbial bile acid metabolism to modify gut microbiota–bile acid interactions	*↑ Lactobacillus and Bifidobacterium; ↓ Bacteroides; ↑* primary/secondary bile acid ratio and unconjugated bile acids	Improve metabolic function

↑ means an increase and ↓ means a decrease.

Physical exercise influences the composition of the intestinal microbiome, alters the functional activity of the gut ecosystem, increases biodiversity, and improves metabolic functions (204). Engaging in physical activity is associated with a higher likelihood of healthy aging. Emerging research suggests that exercise may alter the composition of gut microbiota and their metabolites, leading to health benefits and slowing of disease progression (205). Butyrate, a short-chain fatty acid, supports mitochondrial function and ATP production, which decline with age. Their anti-inflammatory properties reduce oxidative stress and protect mitochondria (206). Butyrate may also slow cellular aging by influencing telomere shortening and by interacting with senescent cells. It induces autophagy, which is essential for cellular maintenance (207, 208). Studies showed that exercise increases the abundance of butyrate-producing bacteria (209–211). Elderly individuals who adhered to physical activity guidelines exhibited a greater presence of advantageous bacteria (such as *Faecalibacterium prausnitzii, Roseburia intestinalis*, and *Lactobacillus*) and a reduced amount of potentially harmful microorganisms, indicating that engaging in physical activity might aid in mitigating age-related gut dysbiosis. Nonetheless, since this was an observational study, it shows an association instead of causation, and the impact of confounding factors like diet and lifestyle cannot be ruled out (212). A sedentary lifestyle has been identified as a risk factor for obesity and various disorders, such as cancer and cardiovascular disease (213). A meta-analysis showed that obese individuals typically engage in more sedentary behaviors and physical inactivity, leading to higher health risks (214). Additionally, a review of 45 reports from various studies showed that high physical activity levels can reduce the risk of several types of cancer by 10%−20%. There is also evidence that physical activity can lower all-cause and cancer-specific mortality by 40–50% in individuals with breast, colorectal, or prostate cancer (215). Although the results suggest that physical exercise may help maintain a healthy gut microbiota in older individuals, it is yet unclear what causes these microbial alterations and whether they are clinically significant.

## 5 Centenarian microbiome: a model to study the relationship between GM and healthy aging

Centenarians, known for their resilience to age-associated diseases, have been studying regarding their gut microbiota in research on healthy aging. Centenarians are regarded as a model of extreme aging to understand the connection between gut bacteria, healthy aging, and longevity.

The structure and function of centenarian GMs exhibit peculiar and functionally resilient characteristics compared to those of ordinary adults (216–219). Generally, centenarian guts are characterized by decreased alpha diversity, reduced levels of butyrate-producing bacteria, and increased mucin degradation by *Akkermansia muciniphila* (220, 221). Sepp et al. (222) analyzed the GM and dietary habits of centenarians (born before 1920), the oldest-old, and young people (born in the late 1990s). Centenarians exhibited higher microbial diversity than young people, frequently dominated by *Prevotella*, which was linked to diets rich in fiber and polyphenol. Their microbiota also comprises environmental microorganisms, such as *Clostridium sensu stricto*, which might affect mood and cognition through serotonin, and *Methanobrevibacter smithii*, which plays a role in fermentation. In comparison, younger people following animal-based diets exhibit a prevalence of *Bacteroides* and butyrate-producing bacteria, such as *Faecalibacterium prausnitzii*, contributing to gut health and anti-inflammatory benefits. Another study in Korea demonstrated that centenarians possessed higher levels of Firmicutes and lower levels of Bacteroidetes than people living in urban areas. Shifts in the microbiota associated with aging featured decreased *Faecalibacterium* and *Prevotella*, and increased levels of *Escherichia, Akkermansia muciniphila, Clostridium*, and *Collinsella*. Variations between independent and hospitalized centenarians were associated with diet and surroundings, with immune-related microbiota pathways being notably active in centenarians (223). In addition, a study in Italy discovered that, while young and elderly people had similar GMs, centenarians had a distinct profile. They had fewer carbohydrate-degrading bacteria such as *Faecalibacterium prausnitzii* and *Eubacterium rectale*, but higher levels of *Methanobrevibacter smithii* and *Bifidobacterium adolescentis*. The gut microbiota of centenarians showed increased glycolysis and fermentation with more genes involved in sugar transport and SCFA production. SCFAs are important for gut health and may play a role in longevity (224).

Centenarians typically have a distinct GM composition. They exhibit lower levels of beneficial bacteria, such as *Faecalibacterium spp*., which are commonly found in younger individuals. Conversely, they show higher levels of health-promoting bacteria such as *Akkermansia spp.*, as well as increased levels of disease-associated pathobionts (225, 226). Although there are concerns regarding the generalizability of these findings, studies on extreme aging have consistently shown similar patterns in GM. Researchers have focused on centenarian groups to identify the distinctive GM signatures that may contribute to longevity and healthy aging. Overall, data on alterations in the gut microbiota in centenarians remain conflicting and may differ based on host genetics, geographic and social factors, diet, lifestyle, demographic characteristics, and methodological variations, like sequencing platforms. The interpretation of these data is further constrained by the paucity of longitudinal investigations, comparatively small sample sizes, and frequent reliance on cross-sectional methods. Therefore, to identify the microbial characteristics consistently linked to healthy longevity, standardized longitudinal studies incorporating metagenomics, metabolomics, and thorough host phenotyping are imperatives.

## 6 Conclusion

The reviewed evidences suggest the gut microbiome's functions in immunological function, metabolic balance, and intestinal barrier integrity as important regulators of healthy aging. Age-related disorders are increasingly linked to gut dysbiosis, which is characterized by decreased microbial diversity, loss of helpful bacteria that produce short-chain fatty acids, and increased intestinal permeability. Notably, the distinctive gut flora of centenarians, enriched in anti-inflammatory and useful SCFAs producing taxa, offers valuable insights into the microbial markers and mechanisms underlying healthy aging and longevity. However, it remains unclear whether the changes in gut microbiota linked to aging are causes of aging or results of age-related physiological changes. Despite significant advances in understanding microbiome-related mechanisms, much of the existing evidence is correlational and based on cross-sectional studies, which restricts causal conclusions. Studies involving human clinical trials and Mendelian randomization that unveil the causal role of the gut microbiome on aging are still limited. Additionally, translating results from animal studies to human contexts is challenging due to variations in host physiology, immune system complexity, and environmental influences. Current clinical trials are limited by small sample size, short follow-up durations, and differences in study design and intervention results. Filling this gap through longitudinal, mechanistic, and causal inference studies will be essential for transforming microbiome research into effective strategies for promoting healthy aging. Microbiome-targeted interventions, including diet and exercise, probiotics, prebiotics, synbiotics, postbiotics, fecal microbiota transplantation, caloric restriction, intermittent fasting, and pharmacological agents, demonstrate significant potential for reestablishing microbial balance and encouraging healthy aging. However, their clinical efficacy remains heterogeneous and context-dependent, differing based on the intervention type, dosage, duration, formulation, and specific microbiome traits, while long-term safety data remain limited. Future studies should focus on longitudinal cohort studies, well-designed randomized controlled trials, causal inference methods (like Mendelian randomization), and multi-omics mechanistic research to differentiate causation from correlation and discover reliable microbiome biomarkers. To progress in this area, a transition toward longitudinal, mechanism-focused, and precision medicine methods is essential, allowing for personalized interventions aimed at the microbiome according to peculiar microbial profiles, while guaranteeing sustained safety, effectiveness, and successful clinical application for healthy aging.
